# Engineering *E. coli* strain for conversion of short chain fatty acids to bioalcohols

**DOI:** 10.1186/1754-6834-6-128

**Published:** 2013-09-10

**Authors:** Anu Jose Mattam, Syed Shams Yazdani

**Affiliations:** 1 Synthetic Biology and Biofuels Group, International Centre for Genetic Engineering and Biotechnology (ICGEB), Aruna Asaf Ali Marg, 110067 New Delhi, India

**Keywords:** Engineered *E. coli*, *Clostridium acetobutylicum*, Butyric acid, Butanol, Fermentation

## Abstract

**Background:**

Recent progress in production of various biofuel precursors and molecules, such as fatty acids, alcohols and alka(e)nes, is a significant step forward for replacing the fossil fuels with renewable fuels. A two-step process, where fatty acids from sugars are produced in the first step and then converted to corresponding biofuel molecules in the second step, seems more viable and attractive at this stage. We have engineered an *Escherichia coli* strain to take care of the second step for converting short chain fatty acids into corresponding alcohols by using butyrate kinase (Buk), phosphotransbutyrylase (Ptb) and aldehyde/alcohol dehydrogenase (AdhE2) from *Clostridium acetobutylicum.*

**Results:**

The engineered *E. coli* was able to convert butyric acid and other short chain fatty acids of chain length C3 to C7 into corresponding alcohols and the efficiency of conversion varied with different *E. coli* strain type. Glycerol proved to be a better donor of ATP and electron as compared to glucose for converting butyric acid to butanol. The engineered *E. coli* was able to tolerate up to 100 mM butyric acid and produced butanol with the conversion rate close to 100% under anaerobic condition. Deletion of native genes, such as fumarate reductase (*frdA*) and alcohol dehydrogenase (*adhE*), responsible for side products succinate and ethanol, which act as electron sink and could compete with butyric acid uptake, did not improve the butanol production efficiency. Indigenous acyl-CoA synthetase (*fadD*) was found to play no role in the conversion of butyric acid to butanol. Engineered *E. coli* was cultivated in a bioreactor under controlled condition where 60 mM butanol was produced within 24 h of cultivation. A continuous bioreactor with the provision of cell recycling allowed the continuous production of butanol at the average productivity of 7.6 mmol/l/h until 240 h.

**Conclusions:**

*E. coli* engineered with the pathway from *C. acetobutylicum* could efficiently convert butyric acid to butanol. Other short chain fatty acids with the chain length of C3 to C7 were also converted to the corresponding alcohols. The ability of engineered strain to convert butyric acid to butanol continuously demonstrates commercial significance of the system.

## Background

Finding different means for production of biofuel molecules will help in gradual shift from usage of fossil fuels [1]. Ethanol so far has served the purpose of alternative fuel due to its easy and cost effective manufacturing process [2]. Butanol, however, is considered to be closer to the fossil fuel in terms of its energy density and hygroscopicity [3-5].

n-Butanol had traditionally been produced by *Clostridium acetobutylicum* through ABE fermentation [4]. *C. acetobutylicum* undergoes acidogenic phase, when it produces majorly acetic acid and butyric acid, followed by solventogenic phase, when it produces acetone, butanol and ethanol (ABE) mix [6]. There are several challenges to the ABE fermentation that prevented this technology from being commercially viable. Some of these challenges include high feedstock cost, low butanol titer, low butanol productivity and strain instability [4,7]. Therefore, engineering efforts have been made to construct non-native industry-friendly host to produce n-butanol. Here, butanol producing pathway from Clostridium sp. has been engineered in laboratory host for heterologous butanol production [8-11]. Further enhancement in butanol yield was made by replacing the pathway intermediate enzyme from the non-Clostridium host [12].

Research recently has been focused on separating alcohol or hydrocarbon production into two distinct biological events - fatty acids production in the first biological event and conversion of the fatty acids into various biofuel molecules in the second biological event. *E. coli* and *Clostridium* species have been used as the common host to fulfill these two functions. Free fatty acid production in engineered *E. coli* has been reported up to 0.3-0.4 g/g glucose [13-15], while native Clostridium was shown to produce 0.45-0.54 g short chain fatty acid per gram of sugar [16,17]. Further, the long chain fatty acids were converted into alcohols and alka(e)nes by the engineered *E. coli*[18,19] and short chain fatty acids were converted into alcohols using *Clostridium* sp. [20-22]. While pathway for independent biological conversion of long chain fatty acids to alcohol or alkane is well characterized and has been successfully used in the heterologous system, the pathway responsible for conversion of short chain fatty acids of C3-C7 length into either alcohol or alkane has been poorly characterized and has not been used in the heterologous system for such purpose. Moreover, native *Clostridium* host used for converting butyric acid to butanol needs to be constantly activated and regenerated through heat-shock and re-inoculation [20], thereby demanding development of more robust, industry friendly platform for this purpose.

Here, we report characterization of *C. acetobutylicum* pathway for conversion of short chain fatty acids into corresponding alcohols and successful engineering of the *E. coli* strain for performing this function. We further show that the yield of conversion is strain specific and internal *E. coli* enzymes do not play a significant role in this process. Most importantly, the process has been validated at the bioreactor level under controlled environment and the engineered *E. coli* cells have been used for continuous production of butanol.

## Results and discussion

### Construction of *E. coli* strain for conversion of butyric acid to butanol

**Figure 1:**
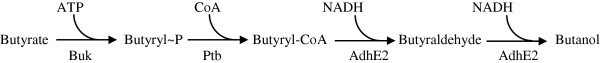
**Metabolic pathway of *Clostridium acetobutylicum *engineered in *E. coli*.** Abbreviations: Buk – butyrate kinase, Ptb – phosphotransbutyrylase, AdhE2 – aldehyde-alcohol dehydrogenase.

*Clostridium acetobutylicum* is known to have an efficient pathway for production of butyric acid in the acidogenic phase as well as conversion of butyric acid to butanol in the solventogenic phase [23]. The production of butyric acid from butyryl-CoA during acidogenic phase happens through a reversible pathway consisting of two enzymes, i.e., butyrate kinase (Buk) and phosphotransbutyrylase (Ptb) [24], while conversion of butyric acid to butyryl-CoA during solventogenic phase occurs through CoA transferase (CoAT) enzyme with concurrent conversion of acetoacetyl-CoA to acetoacetate [25]. Reversal of Buk-Ptb pathway for conversion of butyric acid to the intermediate butyryl-CoA is a more energy efficient process as compared to the equivalent β-oxidation pathway in *E. coli* for exogenous fatty acid activation and their subsequent degradation because clostridial pathway needs one ATP as against requirement of two ATP equivalence for *E. coli* acyl-CoA synthetase (FadD) based pathway [26]. Further, conversion of butyryl-CoA to butanol is more efficiently done by alcohol dehydrogenase from *C. acetobutylicum* as compared to the corresponding native enzyme of host *E. coli* due to its higher affinity towards the butyryl-CoA than the acetyl-CoA [9,27]. Therefore, three genes from *Clostridium acetobutylicum*, i.e., an operon containing phosphotransbutyrylase (*ptb*) and butyrate kinase (*buk*) genes and aldehyde-alcohol dehydrogenase (*adhE2*) gene (Figure 1), which could be used for butyric acid to butanol conversion, were cloned in pQE30 vector and expressed in *E. coli*.

**Figure 2:**
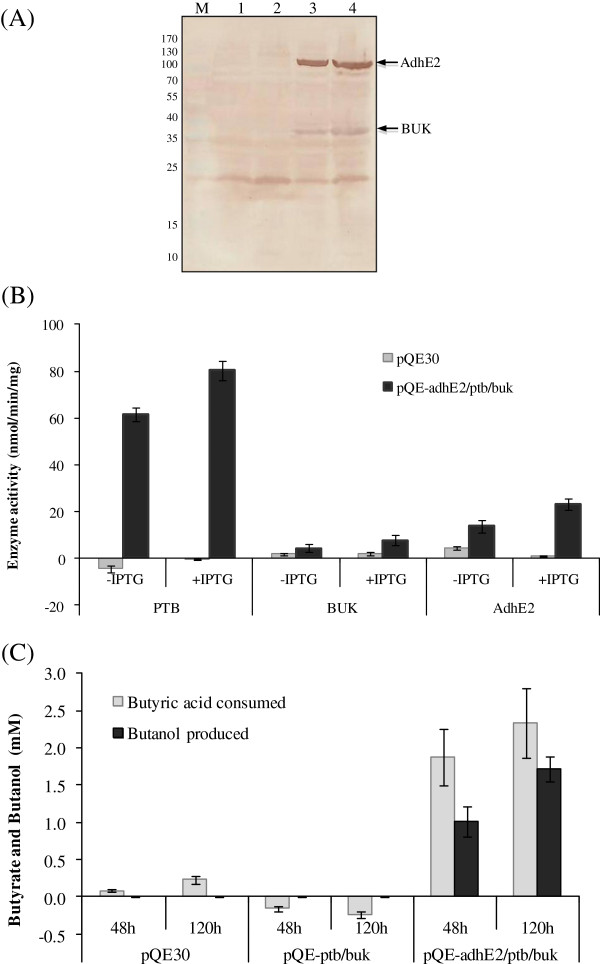
**Expression of clostridial pathway enzymes in *E. coli *for conversion of butyric acid to butanol. (A)** The DH5α strain containing test and control plasmids were grown in LB medium in presence or absence of IPTG and analyzed for the expression of aldehyde/alcohol dehydrogenase (AdhE2) and butyrate kinase (Buk) containing 6-histidine tag on Western blot. Lane M – Molecular weight marker; lane 1 – pQE30 – IPTG; lane 2 - pQE30 + IPTG; lane 3 – pQE-adhE2/ptb/buk – IPTG; lane 4 – pQE-adhE2/ptb/buk + IPTG. **(B)** The grown cells in the LB medium were permeabilized with chloroform and analyzed for the activity of phosphotransbutyrylase (Ptb), Buk and AdhE2. **(C)** Cells containing control and test plasmids were grown in LB medium containing 10 mM butyric acid and samples were withdrawn after 48 h and 120 h to test for butanol production.

The heterologous expression of clostridial genes was tested in the engineered *E. coli* by Western blotting and enzyme assay. Western blotting was performed to assess the expression of two enzymes, Buk (whose gene was placed at the 3’end of *ptb-buk* operon) and AdhE2, where 6-histidine tag was incorporated during cloning as mentioned in the Methods section. Clear bands corresponding to the molecular weight of Buk (~39 kDa) and AdhE2 (~96 kDa) were observed on Western blot using antibody against 6-histidine tag (Figure 2A), indicating their efficient expression in *E. coli*[27,28]. The assay performed to assess enzyme activities of Ptb, Buk and AdhE2 showed 80 nmol/min/mg, 8 nmol/min/mg and 23 nmol/min/mg of activities, respectively (Figure 2B). These values were largely close to the earlier reported enzyme activities for Ptb, Buk and AdhE2 in *E. coli* though we did observe variation in activities perhaps due to different cultivation condition, vector copy number and design of synthetic operon [24,29]. Significant enzyme expression and their activities were also observed in the uninduced cells, suggesting the leaking expression of these enzymes.

Conversion of butyric acid to butanol by the engineered *E. coli* (pQE-adhE2/ptb/buk) strain was tested with relevant controls. Neither pQE30 nor pQE-ptb/buk bearing cells could utilize butyric acid to produce butanol, suggesting all the three clostridial enzymes were needed to convert butyric acid to butanol (Figure 2C). The *E. coli* (pQE-adhE2/ptb/buk) strain produced 1.7 mM butanol from 2.3 mM butyric acid after 120 h of incubation under anaerobic condition. Interestingly, we observed that butyrate concentration in the *E. coli* (pQE – ptb/buk) strain increased when grown for 48 and 120 hours, leading to negative values for butyric acid consumption in Figure 2C. This could be because both Ptb and Buk enzymes were reversible in nature and might be diverting some of the internal butyryl CoA pool of *E. coli* into butyrate.

### Butyric acid tolerance and substrate preference of engineered *E. coli*

**Figure 3:**
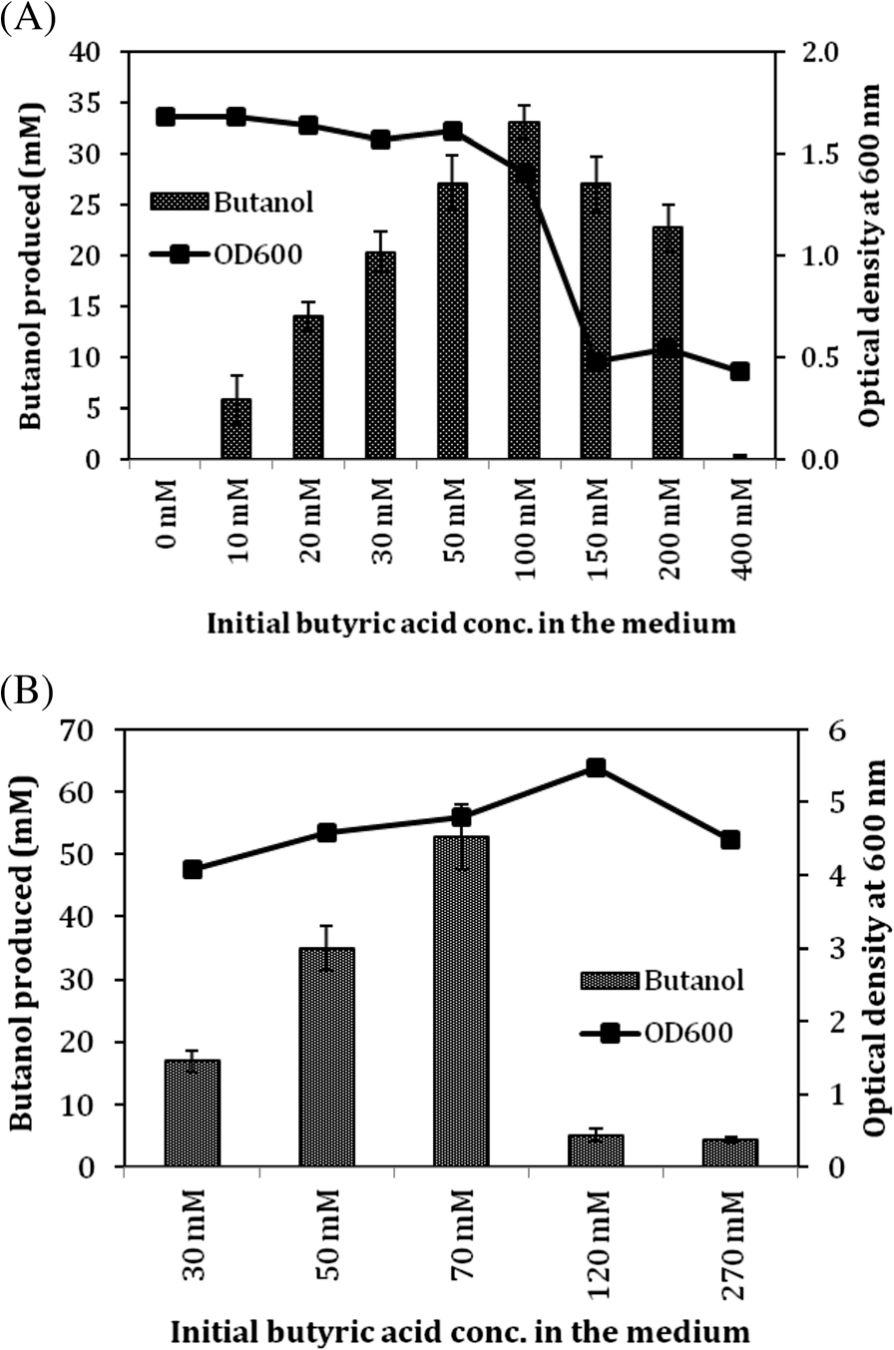
**Butyrate tolerance level of engineered *E. coli*.** The MG1655 strain containing pQE-adhE2/ptb/buk plasmid was grown under anaerobic condition and resuspended in TB medium containing various concentration of butyric acid and 100 mM glycerol to achieve OD_600_ of either 1 **(A)** or 5 **(B)**. The butanol production and cell density were monitored after 120 h of growth in the sealed bottle under anaerobic condition.

For conversion of butyric acid to butanol, it was necessary to investigate the tolerance level of butyric acid to the *E. coli* host strain. We found that butyric acid concentration beyond 100 mM was inhibitory to both cell growth and butanol production (Figure 3A). Four fold higher cell density at the time of induction and butyric acid addition did not improve the tolerance level beyond 100 mM (Figure 3B), though the conversion yield was higher with butanol concentration reaching to 53 mM as against 33 mM for lower cell density. At every concentration of butyric acid tested, there was some butyric acid remained unutilized at the end of cultivation (Additional file 1: Table S1). This observation highlighted need for in-depth study for transport mechanism of butyric acid in *E. coli*.

**Figure 4:**
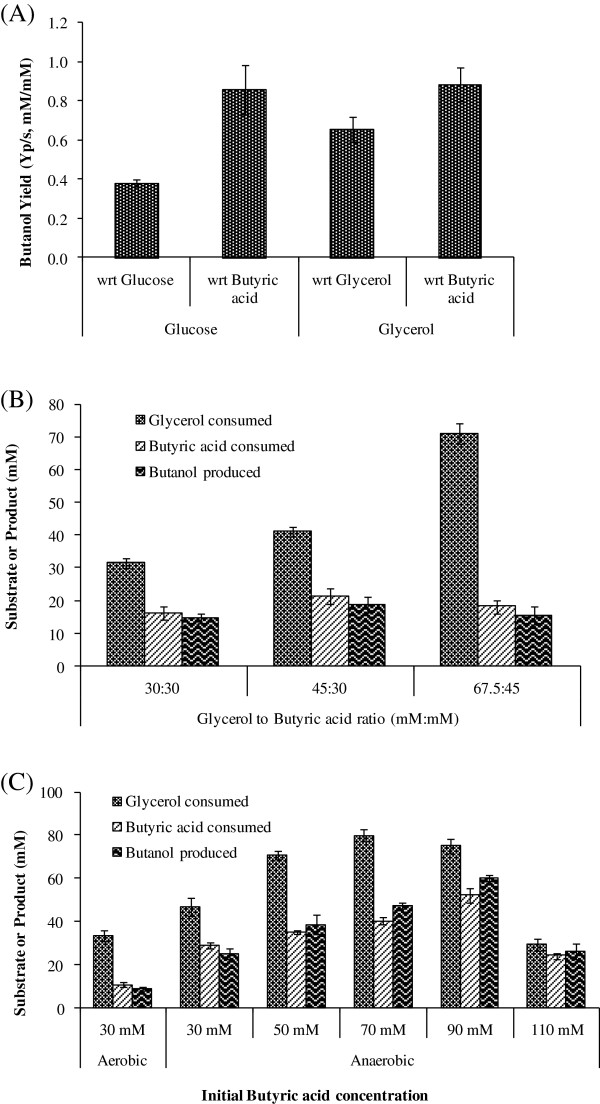
**Substrate specificity and substrate ratio for butanol production. (A)** Impact of electron donor on butanol yield. Engineered *E. coli* MG1655 (pQE-adhE2/ptb/buk) strain was grown under anaerobic condition and resuspended in Terrific Broth with 40 mM butyric acid and 40 mM of either glucose or glycerol as electron donor. Various substrates consumed and butanol produced were analyzed through HPLC after 120 h of incubation. The butanol yield was calculated with respect to (wrt) each carbon source. **(B)** Different ratios of glycerol and butyric acid were tested for production of butanol using cells at the OD_600_ of 1.0. **(C)** Different butyric acid concentrations were tested for production of butanol using cells at the OD_600_ of 10 by keeping the glycerol to butyric acid ratio fixed at 1.5:1.

Butanol production from butyric acid would need one ATP and two NADH (Figure 1). Therefore an optimal electron donor that could satisfy both the requirements needed to be identified. We compared glucose and glycerol as energy and electron source for butanol production. While butanol yield with respect to butyric acid was similar (>85% of theoretical maxima) for either of the substrate, butanol yield with respect to glycerol was approximately doubled as compared to that of glucose (Figure 4A). We further tested butyric acid uptake and butanol production kinetics with respect to various ratios of glycerol and butyric acid. Two ratios of glycerol and butyric acid, 1:1 and 1.5:1, were tested at three different concentrations (Figure 4B). Maximum butanol concentration was obtained when glycerol to butyric acid ratio was 45:30 (mM:mM).

Since glycerol to butyric acid ratio of 1.5:1 worked best for butanol production, we analyzed the effect of increased biocatalyst on butanol production when higher amount of substrates in the same ratio was used. The experiments were performed using biomass with optical density at 600 nm of 10. With this cell density, we first tested impact of growing cells under aerobic vs anaerobic condition before resuspending the culture at OD_600_ of 10 and shifting to anaerobic condition for conversion of butyric acid to butanol. Aerobic cultivation will help achieving the higher cell density faster and therefore will save significant time. However, the results indicated that butanol production was three fold lower when cells were grown under aerobic condition as compared to those grown under anaerobic condition (Figure 4C). We further tested higher concentration of butyric acid, ranging from 50 mM to 110 mM, in the culture media by growing cells under anaerobic condition. Maximum butanol concentration of ~60 mM was obtained when butyric acid concentration in the medium was 90 mM (Figure 4C).

### Conversion of other short chain fatty acids to alcohols

**Figure 5:**
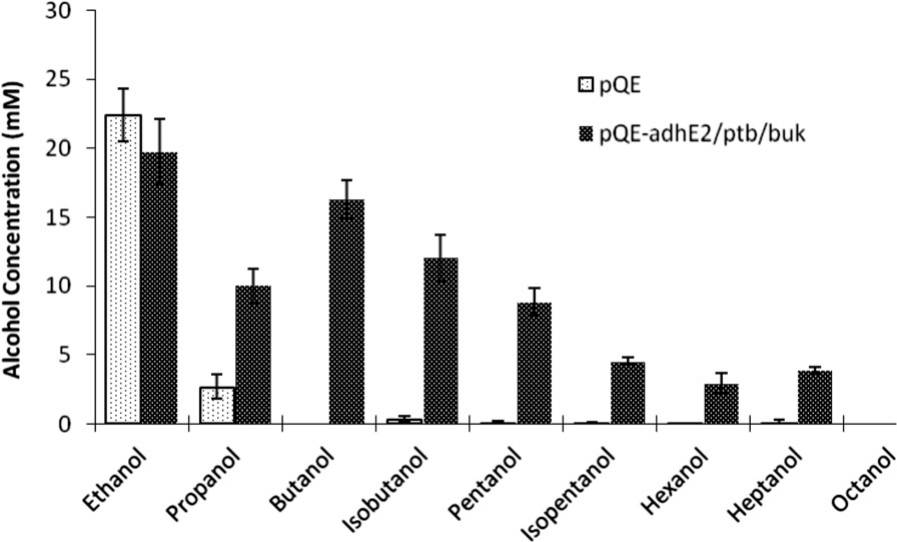
**Substrate specificity of engineered cells towards various short chain fatty acids.** Various short chain fatty acids were added in the growth medium (i.e. Terrific broth + 45 mM glycerol) of *E. coli* MG1655 carrying control or the test plasmid and their conversion to the corresponding alcohol were monitored through HPLC or GC.

We tested the ability of the engineered strain for conversion of other short chain fatty acids of chain length C2-C8 to their corresponding alcohols. We considered acetic acid, propionic acid, butyric acid, isobutyric acid, pentanoic acid, isopentanoic acid, hexanoic acid, heptanoic acid and octanoic acid in our study. The engineered *E. coli* cells containing the clostridial pathway converted all the fatty acids, except octanoic acid, into their corresponding alcohols (Figure 5). The control *E. coli* cells with empty plasmid only converted acetic acid and to a certain extent propionic acid into their corresponding alcohols. The yield of conversion by the engineered *E. coli* varied with chain length as C4 > C3 > C5 > C6 = C7 > C8. The conversion yield was higher with the linear chain fatty acids as compared to the branched chain fatty acids (Figure 5, Additional file 1: Table S2).

### Strain specificity for production of butanol

**Figure 6:**
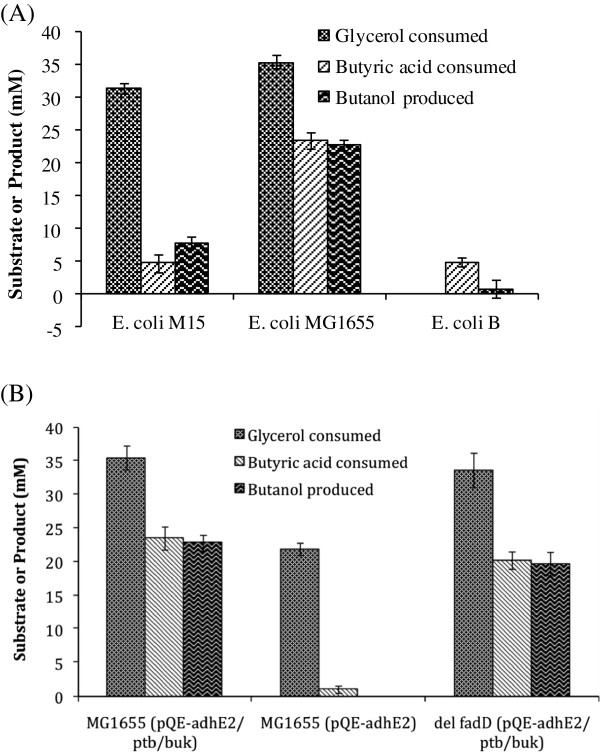
**Strain specificity of engineered cells on the uptake of butyric acid and production of butanol.** Effect of strain type **(A)** and *fadD* gene deletion **(B)** on butyric acid uptake and butanol production was monitored by growing the strains anaerobically in Terrific broth medium containing 45 mM glycerol and 30 mM butyric acid and determining the metabolites through HPLC or GC.

We tested three commonly used laboratory *E. coli* strains, i.e., *E. coli* M15, *E. coli* MG1655 and *E. coli* B, for their ability to convert butyric acid into butanol when transformed with pQE-adhE2/ptb/buk plasmid. All three strains were grown under similar conditions and analyzed for butanol production. *E. coli* MG1655 was able to produce maximum amount of butanol (23 mM) as compared to *E. coli* M15 (7.8 mM) and *E. coli* B (0.84 mM) (Figure 6A), and therefore was selected for further studies. The results suggest that transport ability of different *E. coli* stains for butyric acid may vary significantly. This observation may also be applicable to other studies where *E. coli* was used to convert long chain fatty acids to either alcohol or alka(e)ne [18,19].

*E. coli* MG1655 strain transformed with butyric acid to butanol pathway produced three products besides butanol, i.e., succinic acid, acetic acid and ethanol, when glycerol and butyric acid were used as substrate. Among these, succinic acid and ethanol were sink for NADH since production of each of these molecules from glycolytic intermediates would need 2 molecules of NADH (Additional file 1: Figure S1). Thus, these two molecules were the major competing products for butanol in terms of NADH requirement. We therefore tested deletion mutants for internal alcohol dehydrogenase (*adhE*) and fumarate reductase (*frdA*) in order to prevent formation of ethanol and succinate, respectively. However, these deletions did not lead to enhancement of butanol production (data not shown).

### Role of internal acyl-CoA synthetase in the conversion of butyric acid to butanol

*E. coli* has an internal enzyme, acyl coenzyme A synthetase, encoded by *fadD* gene that facilitates long chain fatty acids uptake and esterification into CoA thioesters prior to its degradation via β-oxidation or incorporation into phospholipids. This enzyme converts free fatty acids into corresponding acyl-CoA with concomitant hydrolysis of ATP into AMP. We wanted to investigate whether FadD had any role to play in the uptake and conversion of butyric acid to butyryl-CoA. When *E. coli* MG1655 was transformed with only alcohol dehydrogenase (AdhE2) from *C. acetobutylicum*, negligible amount of butyric acid was consumed and no butanol was detected in the medium (Figure 6B), suggesting no indigenous enzyme was helping uptake of butyric acid and its assimilation into butyryl-CoA that could be further channeled through AdhE2 to butanol. Moreover, transformation of pQE-adhE2/ptb/buk plasmid, which contained pathway for conversion of butyric acid to butanol from *C. acetobutylicum*, into *fadD* deleted strain resulted in similar butyric acid uptake and butanol production as that of wild type strain, indicating that FadD had no role to play in converting butyric acid to butanol.

### Butanol production by the engineered *E. coli* in the bioreactor

**Figure 7:**
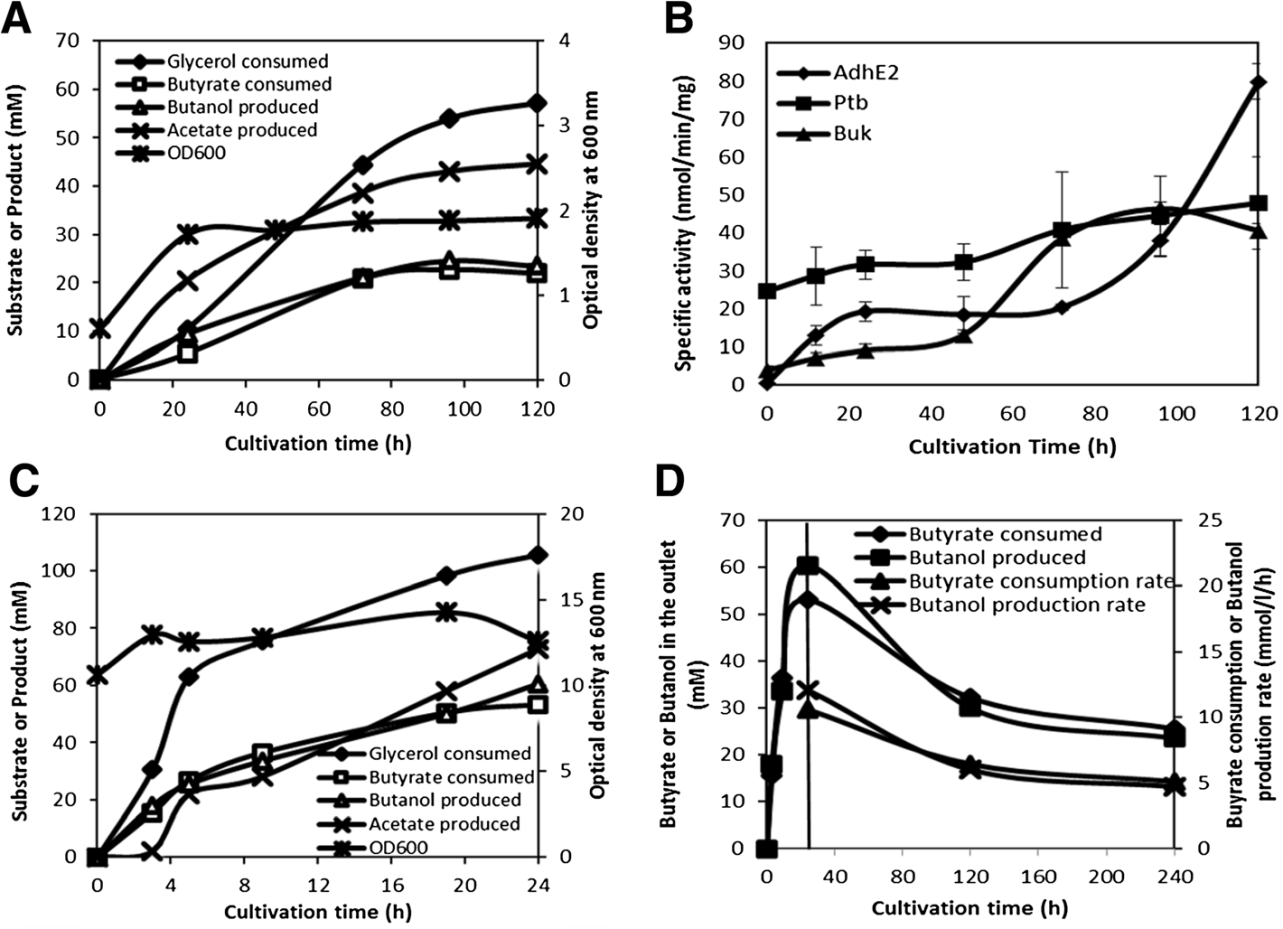
**Production of butanol in the bioreactor in batch and continuous mode. (A)** Fermentation profile and **(B)** enzyme kinetics of the *E. coli* MG1655 (pQE-adhE2/ptb/buk) strain in a bioreactor cultivated in the batch mode with an initial OD_600_ of 1.0. **(C)** Fermentation profile of the engineered strain with an initial OD_600_ of 10. **(D)** Butyric acid consumption and butanol production kinetics in the bioreactor operated under continuous mode with cell recycling using hollow fiber module. Vertical bar at 24 hr indicates the position where fermentation was shifted from batch mode to continuous mode at the dilution rate of 0.2 h^-1^.

Butanol production was analyzed under controlled bioreactor environment, which was necessary to eventually develop a scalable process. Cells were grown under anaerobic condition in the flask, harvested and resuspended in TB medium and grown further in a bioreactor in presence of butyric acid and glycerol as mentioned in the Methods section. The cells produced 25 mM butanol from butyric acid at close to 100% conversion efficiency (Figure 7A). Enzyme kinetic studies during the cultivation indicated consistent production of the three heterologous enzymes in *E. coli*, i.e., Buk, Ptb and AdhE2 (Figure 7B). However, decline in Buk activity, which is the first enzyme in the butyric acid to butanol pathway, towards the end of cultivation might explain decline in flux towards butanol production. Glycerol present in the medium was used by the cells as the source of electron and ATP, which was obvious from the corresponding production of acetate (Figure 7A). To improve the titer and productivity of butanol, we carried out cultivation study in the bioreactor with the starting cell density of OD_600_ ~ 10. The butanol titer reached to 60 mM within 24 hr of fermentation (Figure 7C), as against 25 mM butanol in 100 hr when low cell density was used (Figure 7A).

We further tested continuous production of butanol by cell recycling through hollow-fiber cassette. The feed containing butyric acid and glycerol was started continuously at the rate of 15 mmol/l/h and 22.5 mmol/l/h, respectively, with the dilution rate of 0.2 h^-1^ after 24 hr of fermentation with OD_600_ of 10. The butanol concentration was observed in the permeate intermittently until 240 hrs. An average butanol titer of 37 mM and productivity of 7.6 mmol/l/h was observed in the permeate during the continuous cultivation (Figure 7C). There was a corresponding consumption of butyric acid at the similar rate. Though the butanol production rate was considerably lower than what is expected at the commercial scale, further process development is likely to help in improving the production rate.

## Conclusions

We have shown the efficiency of clostridial pathway in *E. coli* for conversion of short chain fatty acids of chain length C3 – C7 to their corresponding alcohols. We also demonstrated that indigenous enzymes of *E. coli* did not play any significant role in this process. The observation that the conversion could be done in continuous mode using engineered cells as the biocatalysts justifies this process to be further optimized and assessed for commercial application.

## Methods

### Bacterial strains, plasmids and culture conditions

*E. coli* and *C. acetobutylicum* strains used in this study are listed in Table 1. *E. coli* strains were grown at 37°C in Luria–Bertani (LB) medium or Terrific Broth (TB) medium along with 100 μg/ml ampicillin, 30 μg/ml kanamycin and 0.1 mM IPTG as per requirement. All deletion mutant *E. coli* strains used in this study were procured from CGSC (Coli Genetic Stock Centre, Yale University, USA). *E. coli* strains DH5a and M15 were obtained from Invitrogen and Qiagen, respectively. *C. acetobutylicum* ATCC 824 was procured from American Type Culture Collection (ATCC), USA. *C. acetobutylicum* was grown in Reinforced Clostridial Medium (RCM, Himedia Laboratories) in an anaerobic chamber maintained at 37°C. *E. coli* DH5a strain was used for making all the plasmid constructs and pQE30 (Qiagen) was used as expression vector for all the genes. Recombinant DNA techniques were done as per standard procedures [30]. Restriction enzymes and T4 DNA ligase were procured from New England Biolabs (NEB). Plasmid isolation was performed using the kit from Himedia and DNA purification was done using the Sure-Extract PCR Cleanup and Gel Extraction Kit from Genetix. Oligonucleotides to be used as primers were custom synthesized from Sigma-Aldrich. PCR amplification was done using Phusion High Fidelity DNA Polymerase (Finnzymes) and *Taq* Polymerase (Bangalore Genei). All chemicals used in this study were procured from Sigma–Aldrich.

**Table 1:** Strains, plasmids and primers used in this study

**Name**	**Description**	**Reference or source**
**Strains**		
*Clostridium acetobutylicum*		ATCC #824
*E. coli* MG1655	F- LAM- rph-1	CGSC #6300
*E. coli B*	F-	CGSC #5713
*E. coli* DH5α	F- Φ80*lac*ZΔM15 Δ(*lac*ZYA-*arg*F) U169 *rec*A1 *end*A1 *hsd*R17 (rK–, mK+) *pho*A *sup*E44 λ– *thi*^-1^ *gyr*A96 *rel*A1	Invitrogen
*E. coli* M15	F- *thi lac mtl*, pREP4 plasmid	Qiagen
*E. coli* BW25113	rrnB DElacZ4787 HsdR514 DE(araBAD)567 DE(rhaBAD)568 rph-1	CGSC #7636
*E. coli* ΔfadD	BW25113, *ΔfadD :: FRT-kan-FRT*	CGSC #9503
*E. coli* ΔadhE	BW25113, *Δadhe ::FRT-kan-FRT*	CGSC #9113
*E. coli* ΔfrdA	BW25113, *ΔfrdA :: FRT-kan-FRT*	CGSC #10964
**Plasmids**		
pQE30	*bla*, cloning vector	Qiagen
pQE-adhE2	pQE30 with adhe2 gene from *C. acetobutylicum* cloned between BamHI and SalI sites	This study
pQE-ptb/buk	pQE30 with ptb-buk operon from *C. acetobutylicum* cloned between SalI and PstI sites	This study
pQE-adhE2/ptb/buk	pQE-adhE2 with ptb-buk operon from *C. acetobutylicum* cloned between SalI and PstI sites	This study
Primers*		
P1	ATC*GGATCC*ATGAAAGTTACAAATCAAAAA	This study
P2	ACTG*GTCGAC*TTAGTGGTGGTGGTGGTGGTGAAATGATTTTATATAGATATC	This study
P3	ACTG*GTCGAC*GAAGGAGATATACCATGATTAAGAGTTTTAATGAAAT	This study
P4	GT*CTGCAG*TTAGTGGTGGTGGTGGTGGTGTTTGTATTCCTTAGCTTTTTC	This study

*Italicized nucleotides in the primers denote restriction enzyme sites.

Transformed *E. coli* strains were streaked on LB agar plates containing 100 μg/ml ampicillin and/or 30 μg/ml kanamycin and grown overnight at 37°C. Isolated colonies were used to prepare primary inoculum by inoculating in 5 ml LB medium containing antibiotics and growing overnight aerobically. The overnight culture was used to prepare secondary inoculum by inoculating in 20 ml TB medium containing 50 mM glucose or glycerol and incubating overnight at 37°C under anaerobic conditions. The grown secondary inoculum was harvested, resuspended in 20 ml of TB medium to achieve an OD_600_ of 1.0 and transferred in a 100 ml rubber stoppered anaerobic bottle purged with argon gas. The TB medium also contained butyric acid and 0.1 mM IPTG in addition to the appropriate carbon source. The bottle was purged with argon and the culture was grown at 37°C in an orbital shaker. Samples were withdrawn from the bottles at appropriate time points for cell density and metabolite analysis.

The strains were also grown under controlled conditions in a bioreactor. A primary culture was prepared by inoculating an isolated colony in LB and growing the cells overnight at 37°C. The grown culture was used to inoculate 350 ml TB medium in 1 L conical flask containing either glucose or glycerol and incubated for 24 hours in an anaerobic chamber maintained at 37°C. An appropriate volume of this culture was harvested and inoculated in the bioreactor vessel (Multi-vessel BioStat Q Plus fermentor, Sartorius) containing 350 ml of TB medium along with glycerol and butyric acid so as to achieve an initial OD_600_ of 1 or 10. IPTG was not added in the bioreactor as it was shown at the small scale that addition of IPTG had no impact on conversion of butyric acid into butanol (data not shown), perhaps due to sufficient basal level expression of the pathway enzymes. The bioreactor was maintained at 37°C with a stirrer speed of 300 rpm, pH of 7 and purging of highly pure grade argon at a rate of 0.01 L/min to maintain the anaerobic environment.

Continuous production of butanol was achieved by cell recycling using a Hollow Fiber Cartridge (surface area 420 cm^2^, 500,000 NMWC) from GE Healthcare. The cultivation was carried out in a bioreactor with 350 ml working volume under the operating conditions mentioned above with an initial cell density at OD_600_ of 10. Solution containing 50 mM butyric acid, 75 mM glycerol, 72 mM K_2_HPO_4_ and 17 mM KH_2_PO_4_ were added to the fermentor vessel through the peristaltic pump at the dilution rate of 0.2 h^-1^ to achieve feeding of 15 mmol/l/h of butyric acid and 22.5 mmol/l/h of glycerol. Medium along with cells was pumped through the hollow fiber cassette. The cells were recycled back to the bioreactor while permeate from hollow fiber cassette containing butanol was recovered and used for analysis of butanol formation.

### Cloning of *C. acetobutylicum* genes in *E. coli*

Total genomic DNA was isolated from *C. acetobutylicum* ATCC 824 as per standard procedure [30]. The *adhE2* gene of *C. acetobutylicum* was PCR amplified using P1 and P2 primers, digested with *Bam*HI and *Sal*I restriction enzymes and ligated to the corresponding restriction sites of pQE30 plasmid to obtain pQE-adhE2. The P2 primer also contained codons for 6-histidine tag to monitor the expression at the protein level. The *ptb – buk* operon of *C. acetobutylicum* ATCC 824 encoding phosphotransbutyrylase and butyric acid kinase genes was amplified from the genomic DNA using the P3 and P4 primers. The PCR product was digested with *Sal*I and *Pst*I restriction enzymes and ligated to the corresponding restriction sites of pQE30 plasmid to obtain pQE-ptb/buk. P3 primer contained ribosomal biding site for translation initiation and P4 primer contained codons for 6-histidine tag to monitor the expression of operon at the protein level. The *adhE2* gene was further cloned at the *Bam*HI and *Sal*I restriction sites of pQE-ptb/buk plasmid to obtain the final construct pQE-adhE2/ptb/buk.

The expression of the cloned genes was confirmed by SDS – PAGE and Western Blotting. Briefly, the cultures were induced and the crude cell lysates obtained were separated on a 12% polyacrylamide gel. The proteins were transferred to a nitrocellulose membrane (0.2 um, BioTraceNT, Pall Corporation), probed with anti – penta-his antibody (H1029, Sigma) followed by HRP-conjugated anti-mouse IgG antibody (A4416, Sigma). Color development of the blot was performed using DAB (diaminobenzidine) and hydrogen peroxide.

### Analytical methods

For extracellular metabolite analysis, cells were centrifuged at 13000 rpm for 10 min and the supernatant was filtered through 0.22 μm membrane. The extracellular metabolites were analyzed on a 1260 Infinity Series HPLC system (Agilent) equipped with an Aminex HPX-87H anion exchange column (Bio-Rad). Filtered and degassed 4 mM H_2_SO_4_ was used as the mobile phase at a flow rate 0.3 ml/min. The column was maintained at a temperature of 40°C in a thermostat chamber. Butanol was analyzed on a 7890A gas chromatography system (Agilent) with a flame ionization detector equipped with a 7694E headspace analyzer using a HP-5 column (30 m length, 0.32 mm id, 0.25 um film thickness). The oven program was set as follows (40°C for 4 minutes, 80°C for 4 minutes at 10°C/min, 120°C for 2 min at 20°C/min, 200°C for 2 min at 20°C/min). The inlet and detector were maintained at 150°C and 280°C, respectively. Metabolite concentrations were calculated from the area of the curve obtained for 1 g/l of the standards (Absolute Standard). All results were presented as average and standard deviation of the data from two independent experiments.

### Enzyme assays

Phosphotransbutyrylase (Ptb) activity was measured by estimating the liberation of coenzyme A on the addition of butyryl CoA to the reaction mixture. The free coenzyme A formed was then allowed to form a coloured complex with 5,5′ – dithio-(2-nitrobenzoic acid) (DTNB) which could then be estimated by measuring the absorbance at 412 nm [31]. The reaction mixture contained 100 mM potassium phosphate buffer (pH 7.4), 0.02 mM butyryl CoA, 0.08 mM DTNB and crude cell extract. The extinction coefficient of the DTNB – CoA – SH complex was taken as 13.6 mM^-1^ cm^-1^. Aldehyde/alcohol dehydrogenase (AdhE2) activity was measured in the reverse direction by estimating the amount of free NADH liberated during the reaction [32]. Briefly the reaction mixture contained 19 mM sodium pyrophosphate buffer (pH 8.8), 2.37% v/v ethanol or butanol, 5 mM β – NAD and crude cell extract in a total volume of 2 ml. The absorbance at 340 nm was monitored to estimate the amount of free NADH produced. The extinction coefficient of NADH at 340 nm was taken as 6.22 mM^-1^ cm^-1^. The activity of butyrate kinase (Buk) was estimated by monitoring the formation of the coloured ferric hydroxamate complex with butyryl phosphate in the presence of excess of hydroxylamine [33]. The reaction mixture contained 770 mM potassium butyrate (pH 7.5), 48 mM Tris – Cl, 10 mM MgSO4, 700 mM KOH, 10 mM ATP and crude cell extract. The reaction was initiated by the addition of ATP and the mixture was incubated at 37°C for 5 minutes. 1 ml of 10% trichloroacetic acid was used to stop the reaction and the end product was estimated by the addition of 4 ml of FeCl_3_ (1.25% in 1 N HCl). The extinction coefficient of the hydroxamate complex at 540 nm was taken as 0.691 mM^-1^ cm^-1^. The enzyme units were represented as nmol/min/mg.

## Competing interests

The authors declare that they have no competing interests.

## Authors’ contributions

AJM and SSY conceived the study, AJM carried-out all the experiments, AJM and SSY analyzed all the data, SSY drafted the manuscript. All authors read and approved the final manuscript.

## Supplementary Material

Additional file 1: Table S1 Substrate and product concentrations along with conversion yield of butanol with respect to butyrate for all figures. **Table S2:** Concentrations and yield for conversion of short chain fatty acids to alcohols. **Figure S1:** Metabolism of glycerol and mixed acid fermentation pathway of *E. coli*. Genes involved in the pathway - *ldhA* – lactate dehydrogenase, *pflB* – pyruvate formate lyase, *frdABCD* – fumarate reductase, *pta – ack* – phosphotransacetylase and acetate kinase, *adhe* – alcohol dehydrogenase. Glycerol conversion to DHAP is catalyzed by the action of two glycerol dehydrogenases – *glpD* and *glpABC*. Click here for file

## References

[B1] Bhattarai K Stalick WM McKay S Geme G Bhattarai N Biofuel: an alternative to fossil fuel for alleviating world energy and economic crises J Environ Sci Health A Tox Hazard Subst Environ Eng 2011 46 1424 1442 10.1080/10934529.2011.607042 21942396

[B2] Mussatto SI Dragone G Guimarães PM Silva JP Carneiro LM Roberto IC Vicente A Domingues L Teixeira JA Technological trends, global market, and challenges of bio-ethanol production Biotechnol Adv 2010 28 817 830 20630488 10.1016/j.biotechadv.2010.07.001

[B3] Jin C Yao M Liu H Leed CF Ji J Progress in the production and application of n-butanol as a biofuel Renew Sustain Energy Rev 2011 15 4080 4106 10.1016/j.rser.2011.06.001

[B4] Green EM Fermentative production of butanol–the industrial perspective Curr Opin Biotechnol 2011 22 337 343 10.1016/j.copbio.2011.02.004 21367598

[B5] Dürre P Biobutanol: an attractive biofuel Biotechnol J 2007 2 1525 1534 10.1002/biot.200700168 17924389

[B6] Gheshlaghi R Scharer JM Moo-Young M Chou CP Metabolic pathways of clostridia for producing butanol Biotechnol Adv 2009 27 764 781 10.1016/j.biotechadv.2009.06.002 19539744

[B7] Zheng YN Li LZ Xian M Ma YJ Yang JM Xu X He DZ Problems with the microbial production of butanol J Ind Microbiol Biotechnol 2009 36 1127 1138 10.1007/s10295-009-0609-9 19562394

[B8] Atsumi S Cann AF Connor MR Shen CR Smith KM Brynildsen MP Chou KJ Hanai T Liao JC Metabolic engineering of Escherichia coli for 1-butanol production Metab Eng 2008 10 305 311 10.1016/j.ymben.2007.08.003 17942358

[B9] Inui M Suda M Kimura S Yasuda K Suzuki H Toda H Yamamoto S Okino S Suzuki N Yukawa H Expression of Clostridium acetobutylicum butanol synthetic genes in Escherichia coli Appl Microbiol Biotechnol 2008 77 1305 1316 10.1007/s00253-007-1257-5 18060402

[B10] Chen SK Chin WC Tsuge K Huang CC Li SY Fermentation approach for enhancing 1-butanol production using engineered butanologenic Escherichia coli Bioresour Technol 2013 145 204 209 23453982 10.1016/j.biortech.2013.01.115

[B11] Steen EJ Chan R Prasad N Myers S Petzold CJ Redding A Ouellet M Keasling JD Metabolic engineering of Saccharomyces cerevisiae for the production of n-butanol Microb Cell Fact 2008 7 36 10.1186/1475-2859-7-36 19055772 PMC2621116

[B12] Shen CR Lan EI Dekishima Y Baez A Cho KM Liao JC Driving forces enable high-titer anaerobic 1-butanol synthesis in Escherichia coli Appl Environ Microbiol 2011 77 2905 2915 10.1128/AEM.03034-10 21398484 PMC3126405

[B13] Lennen RM Pfleger BF Engineering Escherichia coli to synthesize free fatty acids Trends Biotechnol 2012 30 659 667 10.1016/j.tibtech.2012.09.006 23102412 PMC3856887

[B14] Steen EJ Kang Y Bokinsky G Hu Z Schirmer A McClure A Del Cardayre SB Keasling JD Microbial production of fatty-acid-derived fuels and chemicals from plant biomass Nature 2010 463 559 562 10.1038/nature08721 20111002

[B15] Zhang F Ouellet M Batth TS Adams PD Petzold CJ Mukhopadhyay A Keasling JD Enhancing fatty acid production by the expression of the regulatory transcription factor FadR Metab Eng 2012 14 653 660 10.1016/j.ymben.2012.08.009 23026122

[B16] Zhang C Yang H Yang F Ma Y Current progress on butyric acid production by fermentation Curr Microbiol 2009 59 656 663 10.1007/s00284-009-9491-y 19727942

[B17] Wei D Liu X Yang ST Butyric acid production from sugarcane bagasse hydrolysate by Clostridium tyrobutyricum immobilized in a fibrous-bed bioreactor Bioresour Technol 2013 129 553 560 23270719 10.1016/j.biortech.2012.11.065

[B18] Howard TP Middelhaufe S Moore K Edner C Kolak DM Taylor GN Parker DA Lee R Smirnoff N Aves SJ Love J Synthesis of customized petroleum-replica fuel molecules by targeted modification of free fatty acid pools in *Escherichia coli* Proc Natl Acad Sci U S A 2013 110 7636 7641 10.1073/pnas.1215966110 23610415 PMC3651483

[B19] Akhtar MK Turner NJ Jones PR Carboxylic acid reductase is a versatile enzyme for the conversion of fatty acids into fuels and chemical commodities Proc Natl Acad Sci U S A 2013 110 87 92 10.1073/pnas.1216516110 23248280 PMC3538209

[B20] Richter H Qureshi N Heger S Dien B Cotta MA Angenent LT Prolonged conversion of n-butyrate to n-butanol with Clostridium saccharoperbutylacetonicum in a two-stage continuous culture with in-situ product removal Biotechnol Bioeng 2012 109 913 921 10.1002/bit.24380 22095002

[B21] Baba S Tashiro Y Shinto H Sonomoto K Development of high-speed and highly efficient butanol production systems from butyric acid with high density of living cells of Clostridium saccharoperbutylacetonicum J Biotechnol 2012 157 605 612 10.1016/j.jbiotec.2011.06.004 21683741

[B22] Tashiro Y Shinto H Hayashi M Baba S Kobayashi G Sonomoto K Novel high-efficient butanol production from butyrate by non-growing Clostridium saccharoperbutylacetonicum N1-4 (ATCC 13564) with methyl viologen J Biosci Bioeng 2007 104 238 240 10.1263/jbb.104.238 17964492

[B23] Jurgens G Survase S Berezina O Sklavounos E Linnekoski J Kurkijärvi A Väkevä M van Heiningen A Granström T Butanol production from lignocellulosics Biotechnol Lett 2012 34 1415 1434 10.1007/s10529-012-0926-3 22526420

[B24] Cary JW Petersen DJ Papoutsakis ET Bennett GN Cloning and expression of Clostridium acetobutylicum phosphotransbutyrylase and butyrate kinase genes in Escherichia coli J Bacteriol 1988 170 4613 4618 2844725 10.1128/jb.170.10.4613-4618.1988 PMC211500

[B25] Clark SW Bennett GN Rudolph FB Isolation and characterization of mutants of Clostridium acetobutylicum ATCC 824 deficient in acetoacetyl-Coenzyme A:Acetate/Butyrate:Coenzyme A-Transferase (EC 2.8.3.9) and in Other Solvent Pathway Enzymes Appl Environ Microbiol 1989 55 970 976 16347898 10.1128/aem.55.4.970-976.1989 PMC184233

[B26] Black PN DiRusso CC Metzger AK Heimert TL Cloning, sequencing, and expression of the fadD gene of Escherichia coli encoding acyl coenzyme a synthetase J Biol Chem 1992 267 25513 25520 1460045

[B27] Fontaine L Meynial- Salles I Girbai L Yang X Croux C Soucaille P Molecular characterization and transcriptional analysis of *adhE2*, the gene encoding the NADH – dependent aldehyde/alcohol dehydrogenase responsible for butanol production in alcohologenic cultures of *Clostridium acetobutylicum* ATCC 824 J Bacteriol 2002 184 821 830 10.1128/JB.184.3.821-830.2002 11790753 PMC139506

[B28] Hartmanis MG Butyrate kinase from C*lostridium acetobutylicum* J Biol Chem 1987 262 617 621 3027059

[B29] Berezina OV Zakharova NV Brandt A Yarotsky SV Schwarz WH Zverlov VV Reconstructing the clostridial n-butanol metabolic pathway in *Lactobacillus brevis* Appl Microbiol Biotechnol 2010 87 635 646 10.1007/s00253-010-2480-z 20195860

[B30] Sambrook J Fritsch EF Maniatis T Molecular cloning, a laboratory manual 1989 2 Cold Spring Harbour, NY: Cold Spring Harbor Laboratory Press

[B31] Andersch W Bahl H Gottschalk G Level of enzymes involved in acetate, butyrate, acetone and butanol formation by *Clostridium acetobutylicum* Eur J Appl Microbiol Biotechnol 1983 18 327 333 10.1007/BF00504740

[B32] Kagi J Vallee B The role of zinc in alcohol dehydrogenase: the effect of metal binding agents on the structure of the yeast alcohol dehydrogenase molecule J Biol Chem 1960 235 3188 3192 13750715

[B33] Rose IA Acetate kinase of bacteria (acetokinase) Methods Enzymol 1955 1 591 593

