# Supplementary material for: Engineering E. coli strain for conversion of short chain fatty acids to bioalcohols
Source: Biotechnol Biofuels. 2013 Sep 10;6:128. doi: 10.1186/1754-6834-6-128 (PMC3847231; doi:10.1186/1754-6834-6-128)
Supplement: Additional file 1: Table S1 — Substrate and product concentrations along with conversion yield of butanol with respect to butyrate for all figures. Table S2: Concentrations and yield for conversion of short chain fatty acids to alcohols. Figure S1: Metabolism of glycerol and mixed acid fermentation pathway of E. coli. Genes involved in the pathway - ldhA – lactate dehydrogenase, pflB – pyruvate formate lyase, frdABCD – fumarate reductase, pta – ack – phosphotransacetylase and acetate kinase, adhe – alcohol dehydrogenase. Glycerol conversion to DHAP is catalyzed by the action of two glycerol dehydrogenases – glpD and glpABC. [file 1754-6834-6-128-S1.doc]

**Supplementary Table S1.** Substrate and product concentrations along with conversion yield of butanol with respect to butyrate for all figures

| **Figure No.** | **Strain** | **Glycerol consumed (mM)** | **Residual glycerol (mM)** | **Butyrate consumed (mM)** | **Residual butyrate (mM)** | **Butanol produced (mM)** | **Conversion Yield (mM butanol/mM butyrate consumed)** |
| --- | --- | --- | --- | --- | --- | --- | --- |
| 3A | MG1655 pQE adhE2/ptb/buk | 11.2112 | 3.8742 | 6.176237 | 6.311072 | 5.823334 | 0.942861 |
|  |  | 25.2363 | 5.0217 | 14.05924 | 5.386077 | 13.99885 | 0.995705 |
|  |  | 39.699 | 4.3294 | 20.12462 | 8.314698 | 20.33218 | 1.010314 |
|  |  | 40.2962 | 7.6402 | 27.93928 | 21.64689 | 27.13738 | 0.971299 |
|  |  | 42.93372 | 76.6561 | 34.73171 | 73.33759 | 33.06318 | 0.951959 |
|  |  | 35.38403 | 79.24891 | 11.38805 | 128.4197 | 27.05825 | 2.376021 |
|  |  | 30.56038 | 75.35586 | 12.07007 | 189.0274 | 22.73239 | 1.883368 |
|  |  | 13.71199 | 69.42061 | 24.85079 | 396.3666 | 0.240285 | 0.009669 |
|  |  |  |  |  |  |  |  |
|  |  |  |  |  |  |  |  |
| 3B | MG1655 pQE adhE2/ptb/buk | 28.11465 | 9.471284 | 15.17163 | 14.70865 | 18.05532 | 1.190072 |
|  |  | 63.15549 | 13.19494 | 29.15922 | 16.50848 | 35.79966 | 1.227731 |
|  |  | 133.0163 | 0.708984 | 44.34805 | 19.40911 | 53.19907 | 1.199581 |
|  |  | 37.5012 | 179.9498 | 4.517272 | 114.3943 | 4.90004 | 1.084734 |
|  |  | 17.28655 | 410.4119 | 8.869817 | 226.7752 | 3.966098 | 0.447145 |
|  |  |  |  |  |  |  |  |
|  |  |  |  |  |  |  |  |
| 4B | MG1655 pQE adhE2/ptb/buk | 31.48825 | 16.00818 | 16.07573 | 17.21088 | 14.33952 | 0.891998 |
|  |  | 41.00051 | 12.28616 | 21.31372 | 9.114645 | 18.71696 | 0.878165 |
|  |  | 71.01815 | 2.061607 | 18.02301 | 34.51986 | 15.42202 | 0.855685 |
|  |  |  |  |  |  |  |  |
|  |  |  |  |  |  |  |  |
| 4C | MG1655 pQE adhE2/ptb/buk | 33.34181 | 13.97999 | 10.54548 | 20.0421 | 8.860052 | 0.840176 |
|  |  | 46.73242 | 2.810685 | 28.86429 | 4.2195 | 24.73113 | 0.856807 |
|  |  | 70.74759 | 24.81922 | 35.03602 | 15.43367 | 38.49941 | 1.098852 |
|  |  | 79.82374 | 38.69823 | 40.04159 | 22.23606 | 47.33496 | 1.182145 |
|  |  | 75.04482 | 91.97382 | 52.1932 | 39.63009 | 59.93658 | 1.14836 |
|  |  | 29.09376 | 158.0289 | 24.01114 | 83.36101 | 26.06718 | 1.085629 |
|  |  |  |  |  |  |  |  |
| 6 | M15 pQE adhE2/ptb/buk | 31.49903 | 15.87597 | 4.745248 | 27.09835 | 7.866684 | 1.657803 |
|  | MG1655 pQE adhE2/ptb/buk | 35.44251 | 8.580887 | 23.48449 | 6.247513 | 22.78401 | 0.970173 |
|  | E. coli B pQE adhE2/ptb/buk | 0 | 46.0135 | 4.967603 | 26.3028 | 0.845803 | 0.170264 |
|  |  |  |  |  |  |  |  |
|  |  |  |  |  |  |  |  |
| 7A | MG1655 pQE adhE2/ptb/buk | 57.1542 | 0 | 21.97818 | 19.37515 | 23.47850 | 1.068264 |
|  |  |  |  |  |  |  |  |
|  |  |  |  |  |  |  |  |
| 7B | MG1655 pQE adhE2/ptb/buk | 105.6912 | 52.97488 | 53.16143 | 31.25848 | 60.33334 | 1.134908 |
|  |  |  |  |  |  |  |  |
|  |  |  |  |  |  |  |  |
|  |  |  |  |  |  |  |  |
|  |  |  |  |  |  |  |  |
|  |  |  |  |  |  |  |  |
|  |  |  |  |  |  |  |  |
|  | | | | | | | |

**Supplementary Table S2. Concentrations and yield for conversion of short chain fatty acids to alcohols**

|  |  | | |  |  |  |  |  |  |
| --- | --- | --- | --- | --- | --- | --- | --- | --- | --- |
| **Acid** | | **Glycerol consumed** | | | **Residual Glycerol** | **Fatty acid consumed** | **Residual fatty acid** | **Alcohol produced** | **Conversion Yield (wrt fatty acid consumed)** |
|  | **Acetic acid** | | 35.50492 | | 4.641139 | 22.3028 | 5.72957 | 19.72755 | 0.884532 |
|  | **Propanoic acid** | | 37.26668 | | 5.540624 | 17.939 | 11.0823 | 10.01702 | 0.558393 |
|  | **Butyric acid** | | 40.23534 | | 3.386311 | 14.29439 | 17.52984 | 16.30677 | 1.140781 |
|  | **Isobutyric acid** | | 32.35561 | | 7.467321 | 11.10831 | 17.89169 | 12.06054 | 1.085722 |
|  | **Pentanoic acid** | | 29.48622286 | | 18.2906 | 10.2301 | 17.7879 | 8.80245 | 0.860446 |
|  | **Isopentanoic acid** | | 43.49190409 | | 9.625686 | 3.462265 | 24.83174 | 4.524444 | 1.306787 |
|  | **Hexanoic acid** | | 39.7321 | | 5.2138 | 4.321 | 25.0202 | 2.91 | 0.673455 |
|  | **Heptanoic acid** | | 40.2139 | | 4.0297 | 5.0219 | 25.1911 | 3.853499 | 0.767339 |
|  | **Octanoic acid** | | 37.0321 | | 7.2104 | 0 | 29.384 | 0 | NA |

Succinic Acid

2 NADH

Acetyl CoA

Ethanol

Acetic Acid

2 NADH

*pta - ack*

*adhe*

Pyruvate

Phosphoenolyruvate (PEP)

Dihydroxyacetone phosphate (DHAP)

NADH

NADH

Lactic Acid

NADH

*ldhA*

*pflB*

*frdABCD*

Glycerol

**Supplementary Figure S1.** Metabolism of glycerol and mixed acid fermentation of pathway.

Genes involved in the pathway - *ldhA* – lactate dehydrogenase, *pflB* – pyruvate formate lyase, *frdABCD* – fumarate reductase, *pta – ack* – phosphotranacetylase and acetate kinase, *adhe* – alcohol dehydrogenase. Glycerol conversion to DHAP is catalyzed by the action of two glycerol dehydrogenases – *glpD* and *glpABC*.
